# A simple method to assess group difference in RT-qPCR reference gene selection using GeNorm: The case of the placental sex

**DOI:** 10.1038/s41598-017-16916-y

**Published:** 2017-12-05

**Authors:** Joey St-Pierre, Jean-Charles Grégoire, Cathy Vaillancourt

**Affiliations:** 1 INRS-Centre Institut Armand-Frappier and BioMed Research Center, Laval, Canada; 2 Center for Interdisciplinary Research on Well-Being, Health, Society and Environment, Université du Québec à Montréal, Montréal, QC Canada; 3 INRS-Centre Énergie Matériaux Télécommunications, Montréal, QC Canada

## Abstract

Normalization with proper reference genes is a crucial step in obtaining accurate mRNA expression levels in RT-qPCR experiments. GeNorm and NormFinder are two commonly used software packages that help in selecting the best reference genes, based on their expression stability. However, GeNorm does not take into account a group variable, such as sample sex, in its calculation. We demonstrate a simple calculation step to assess the variability of such parameters by multiplying the GeNorm M value with the difference of Cq values between groups. To test this, we used 28 reference gene candidates, to analyze 20 placental samples (10 of each sex), and by using *HPRT1* (lower Cq values in male placentas (*P* = 0.017)), as a target gene. Our calculation demonstrates that the *RPL30* – *GAPDH* reference gene combination is the better option to assess small placental sex differences in mRNA level, *versus* the selection obtained from GeNorm or NormFinder. The *HPRT1* normalized mRNA expression level is different between placental sexes, using *RPL30* and *GAPDH* as reference genes (*P* = 0.01), but not when using genes suggested by GeNorm or NormFinder. These results indicate that the proposed calculation is appropriate to assess small variations in mRNA expression between 2 groups.

## Introduction

Reverse transcription quantitative polymerase chain reaction (RT-qPCR) is an efficient tool to assess mRNA level. However, the production of reliable results requires several steps to be followed, which has not always been the case in some publications using RT-qPCR results^1,2^. An important step is the selection of stable mRNA reference genes, in order to achieve accurate normalization^3^.

Poor reference gene selection can lead to inaccurate results^4^. Reference genes are often described as housekeeping genes that are necessary for regular cell function. These genes are expected to have a stable mRNA expression level throughout all cell types and conditions tested^5^. However, this is rarely the case as diseases, treatments or experimental conditions affect the stability of commonly used reference genes^6^. This indicates the need to test for reference gene variability between treatments or sample conditions, for all experiments. Several software options are widely utilized to assess the variability of reference genes, including GeNorm and NormFinder for qPCR experiment^3,4^. While NormFinder offers a method of reference genes selection that takes into account intra- and inter-group variability, such as sex, GeNorm does not differentiate between groups of samples or treatments^4,7^.

Sample sex is an important parameter for biological and molecular biology research, with grant funding bodies and publishers requiring sample sex to be evaluated or clear reasons for its exclusion^8–11^. Placental research is an area where fetal sex differences are of significant importance^12–14^, including in regard to placental adaptation strategy^12,15,16^. Unfortunately, placental/fetal sex is not always a universally adopted parameter^8^.

Reference gene selection is especially important when working with tissue samples or primary cell lines. This study demonstrates a new method of calculation for the selection of reference genes when assessing the difference between two groups, exemplified in this case by placental sex.

## Results

### Cq results

**Figure 1:**
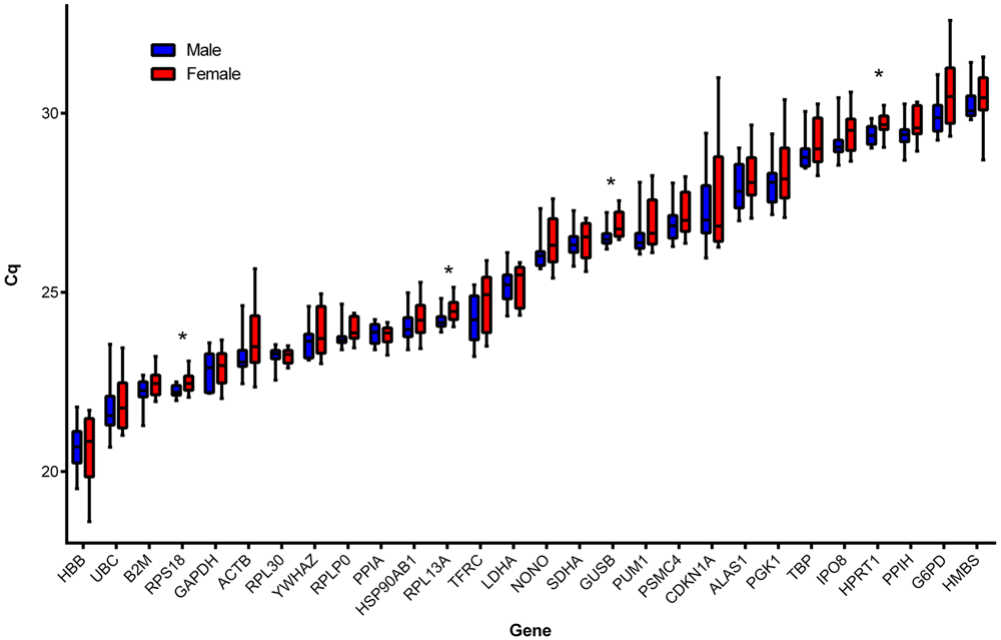
Distribution of the Cq values obtained for 28 candidate reference genes, from the reference genes H96 and H96 plus primePCR plates (Bio-Rad, see Table 1) for 10 placental samples for each fetal sex. Boxes represent the median Cq values with interquartile ranges representing minimum value to maximum value. Student’s T tests were performed on Cq values between placental sexes. *P ≤ 0.05. n = 20 placentas (10 males; 10 females).

Figure 1 shows the distribution of Cq values for 28 reference genes in 20 placental tissues (10 for each sex) from PrimePCR Reference Gene H96 and Reference Gene H96 plus plates. Four reference genes showed significantly lower Cq, indicating higher mRNA levels, in male compared to female placentas (*RPS18*, p = 0.018;* RPL13A*, p = 0.017; *GUSB*, p = 0.015; *HPRT1*, p = 0.017). Results were similar using the absolute quantity of mRNA (2^∆Cq^) (data not shown).

### GeNorm and NormFinder

**Figure 2:**
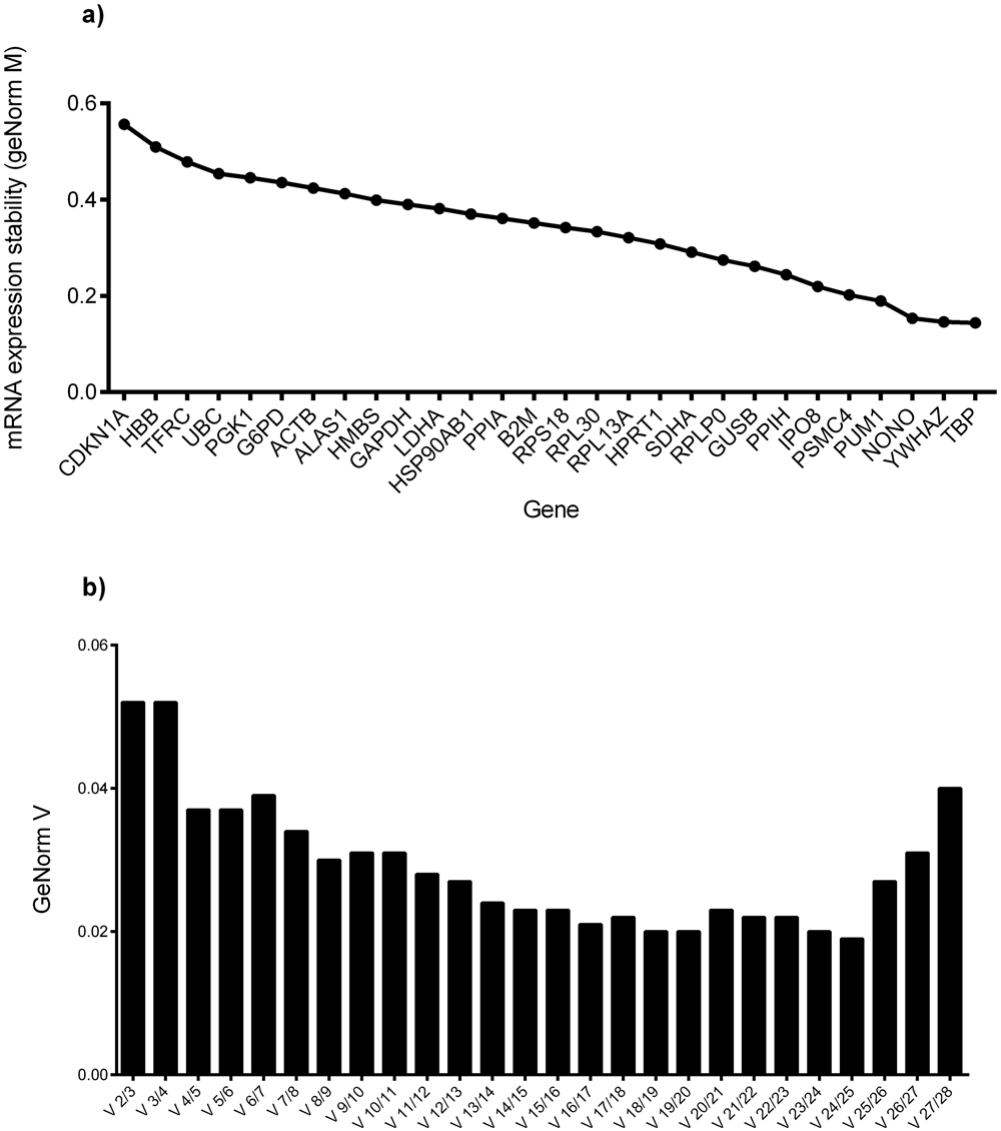
(**a**) GeNorm M results for all reference genes tested for all the placenta samples taken together. (**b**) GeNorm V value for all reference genes tested. These results represent the best reference genes selection (lower GeNorm M score) as well as the quantity of reference genes optimal to use found (GeNorm V score below 0.15) using the QbasePlus software.

Figure 2a shows the GeNorm M analysis for all 20 placental samples. Lower GeNorm M values represent the most stable reference genes. The GeNorm software package also computes variation of reference genes used for normalization (GeNorm V: n/(n + 1), indicating that 2 reference genes are suitable for RT-qPCR normalization in our experiment (2/3 genes GeNorm V < 0.15) (Fig. 2b). Thus, for all 20 samples, the best reference genes pair according to the GeNorm software is *TBP* and *YWHAZ*.

**Figure 3:**
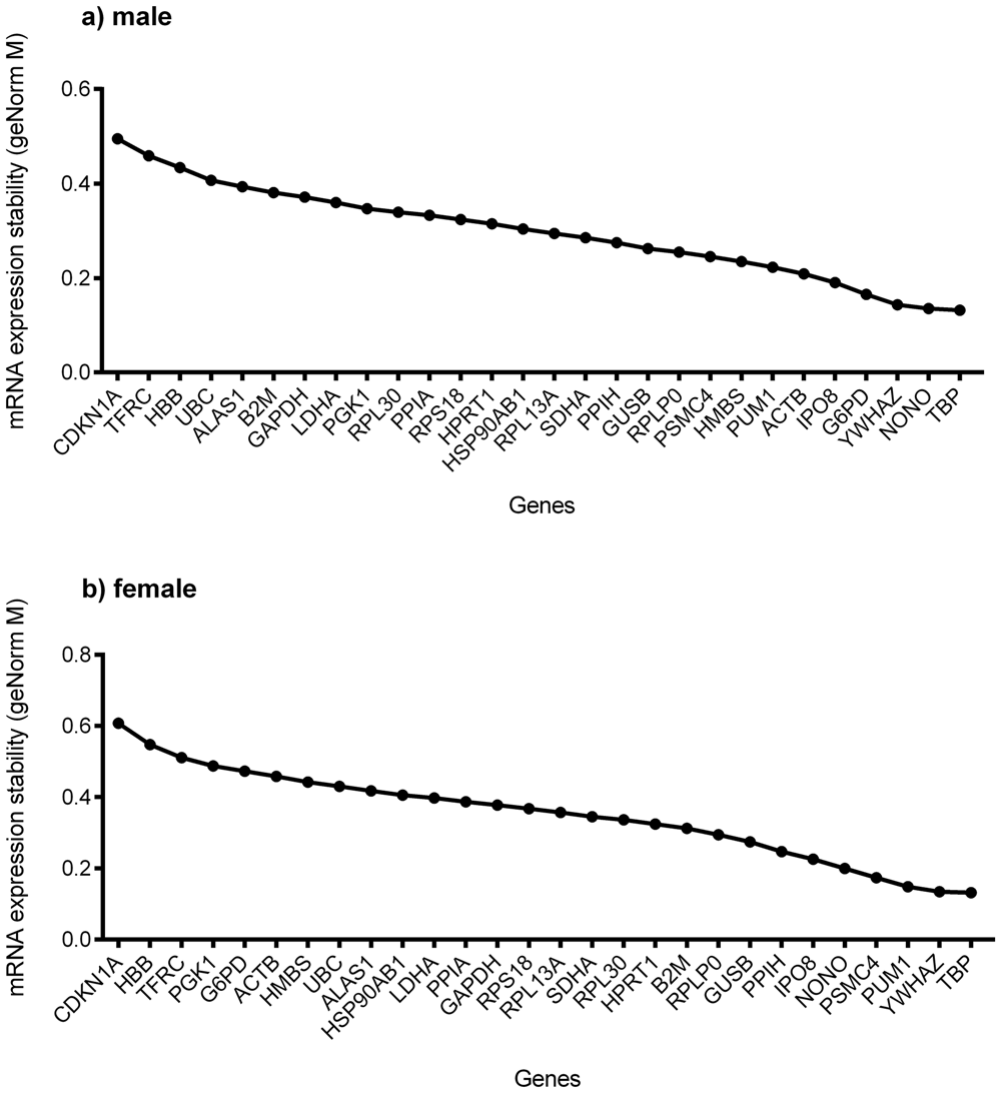
GeNorm results for (**a**) male placentas and (**b**) female placentas. Ten placentas from each sex were used to obtain results with PrimePCR Reference Gene H96 and Reference Gene H96 Plus plates (Bio-Rad). Cq values were transferred into the Qbase Plus software (Biogazelle) to obtain GeNorm M values.

The samples were then split into two sets according to sex, which were entered separately in the GeNorm software package. Results demonstrate that for male placentas, *NONO* and *TBP* are the most stable reference genes (Fig. 3a), whilst for female placentas, *TBP* and *YWHAZ* are the most stable (Fig. 3b). Four genes have a highly different GeNorm rank between the sexes: *G6PD, ACTB, HMBS* and *PGK1*. The reference gene selection software NormFinder was also used to assess the best reference gene using intra- and inter-group (*i.e*. placental sex) variation. Results indicate *IPO8* and *TBP* to be the combination of reference genes to utilize for normalization (supplemental data Figure S1).

### Novel calculation method

**Figure 4:**
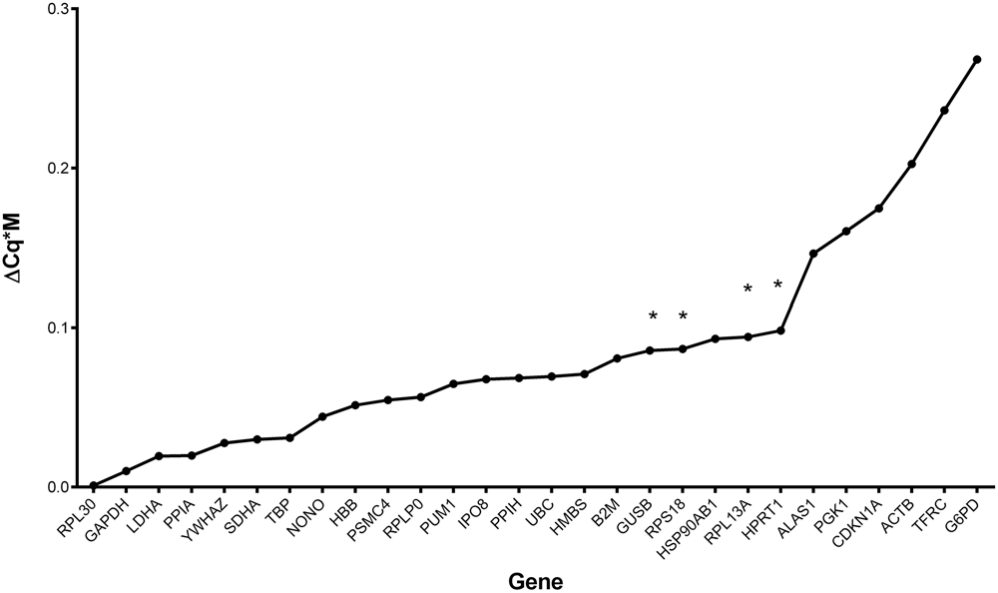
Results from multiplication of the GeNorm M value with the difference of average of Cq results between placental sexes (ΔCq). A smaller ΔCq*M score represents the optimal value for gene expression results normalization for two groups (placental sex). *Genes showing significantly different Cq values between male and female placentas (Student’s T test, P ≤ 0.05; see Fig. 1).

A novel calculation method was used to obtain the best reference genes possible for normalization in the two groups. This calculation requires the multiplication of the GeNorm M value by the difference of Cq between the groups (ΔCq*M). The lowest number indicates the genes for which normalization provides the smallest bias for groups, while still accounting for pairwise variation (Fig. 4). Using this calculation on the 20 samples, the *RPL30* and *GAPDH* pair is the best combination of reference genes.

**Figure 5:**
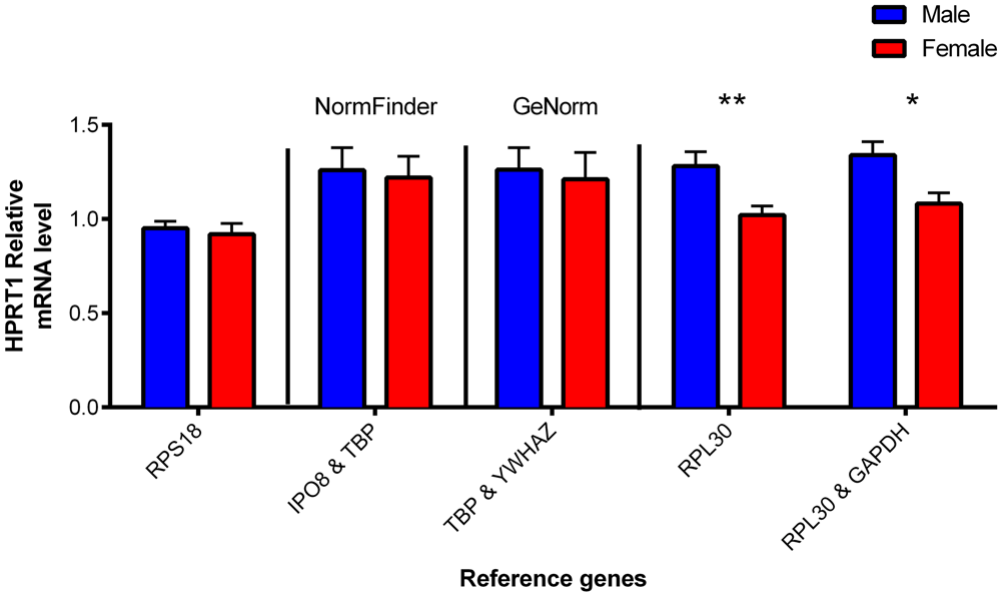
*HPRT1* mRNA expression levels normalized with different reference genes in human placenta. *RPS18* has significantly different Cq values between sexes. *IPO & TBP* were selected using NormFinder software. *TBP & YWHAZ* were selected using GeNorm software. *RPL30* and *RPL30 & GAPDH* were selected using the ΔCq form the placental sex multiplied by GeNorm M value. *p ≤ 0.05; **p ≤ 0.01 n = 10 per placental sex.

To validate this calculation, *HPRT1* was used as a target gene for normalized mRNA level (2^∆∆Cq^), given that *HPRT1* mRNA level is significantly different between males and females. Figure 5 represents the normalized *HPRT1* mRNA level, using different reference genes chosen from the results of GeNorm (Fig. 2) and NormFinder (Fig. S1). *RPS18* was chosen as a reference gene to show normalizing with a reference gene that has a significant difference between two groups. As expected, normalizing with *RPS18* resulted in no significant difference in *HPRT1* mRNA level between sexes (Fig. 5). *IPO8* and *TBP* were selected as best reference genes by NormFinder (Fig. S1), while *TBP* and *YWHAZ* are the two best reference genes obtained with the GeNorm calculation for our samples (Fig. 2a). However, when normalizing with either *IPO8* and *TBP* or *TBP* and *YWHAZ*, there was no significant difference between males and females for placental *HPRT1* mRNA level (Fig. 5). When *RPL30* and the *RPL30*+ *GAPDH* combination were used as reference genes, a significantly higher relative *HPRT1* mRNA level was obtained in male placentas (p = 0.009 and p = 0.010, respectively; Fig. 5). Results were similar for the other genes that have a significantly different absolute mRNA levels between placental sexes (*RPS18*, *GUSB* and *RPL13A* genes, data not shown). However, when normalizing every other gene with the *RPL30* and *GAPDH* combination, no statistically significant difference was evident between male and female placentas (data not shown). This indicates that the new calculation method proposed here for choosing the best reference genes, did not result in “false positive” group differences after normalization.

## Discussion

In this study, we demonstrate the importance of placental sex in reference gene selection and that proper reference genes selection is crucial to obtaining accurate results. The results here show that placental sex needs to be taken into consideration in the selection of reference genes for the normalization of placental mRNA expression levels. Significant Cq differences in placental mRNA levels were evident in some of the most commonly used reference genes: *HPRT1*, *GUSB*, *RPL13A* and *RPS18*. These genes had significantly lower Cq values for male, *versus* female, placentas, indicating that their utilization for gene normalization would produce biased results, both inappropriate non-significant and significant results^1,2,4,17^.

Cvitic *et al*. found that most of the placental mRNA level of genes tested were higher in boy placentas than in girl placentas in microarray analysis, but this varied greatly between cell types^18^. This team used *HPRT1* as reference gene, but in their analysis, *HPRT1* did not vary between samples sexes. *HPRT1* is a gene that is present on the chromosome X. The *HPRT1* gene is inactivated in early embryology in female fetuses and has already been shown to have significantly different expression in regard to sample sex in other tissues^19–21^. In our study, mRNA levels for *GUSB, RPL13A, RPS18* and *HPRT1* are significantly higher in boy placentas than in girl placentas (lower Cq) and we did not observe any reference gene mRNA levels that were higher in girl (as opposed to boy) placentas. These could be explained by the fact that the RNA taken in our study was obtained from the trophoblastic layer of the placenta that has a generally higher overall gene expression in male placentas compared to female placentas^14,18^.

It is noteworthy that *YWHAZ* was seen expressed significantly higher in male placentas compared to female placentas by Cleal *et al*.^14^ but not in our study. This could be explained by the fact that the authors tested 102 placentas compared to 20 placentas from our study, perhaps leading to a sampling bias resulting from less placental samples^14^. Another difference between their study compared to ours is that the sampling method might be different as the method used by Cleal *et al*. is not specified. However, the inconsistency between Cleal *et al*. results and ours highlights the importance to test and select reference genes for their difference in Cq values for every sample group in every experiment and not to rely only on previous literature.

Choosing a reference gene that does not vary between groups is essential for accurate RT-qPCR results, and this can be applied to placental sex as a subgroup^7^. In this study, we tested 28 different candidate reference genes for 20 human placental samples and rated them from best to worst using two of the most popular methods. Yet, when normalizing with the reference genes combinations found by commonly used software packages, there was no significant difference in *HPRT1* mRNA level between male and female placentas. This would suggest that NormFinder’s reference gene selection does not include proper correction for the difference in the average gene expression, at least in our experiment.

When using reference genes selected by our new method of calculation, we observed a significant difference in the placental mRNA level of *HPRT1* between male and female placentas, that reflects the significant difference observed for the Cq values and for the absolute mRNA expression. The same is also observed for all genes which have significantly different Cq values between male and female populations. Moreover, using our calculation and in accordance with the Cq values obtained, we did not identify other significant differences in mRNA levels between sexes other than those that were significantly different for their Cq values and absolute mRNA level (*GUSB, RPL13A* and *RPS18*). This would mean that, when comparing for different groups (or in our case placental sexes), multiplying GeNorm M results by the Cq difference between groups will enable to find reference genes that will provide a more accurate normalization for group comparison, while not obtaining false-positive results.

However, this simple tool should be used with caution and does not replace a researcher’s judgement, as is the case with any other method of selecting reference genes for RT-qPCR. Furthermore, the calculations we propose might not fit all situations encountered as they have only been tested human placentas and only for groups based on fetal sexes.

## Conclusion

The main finding in this study is that even though the genes with the best GeNorm M values could be used to normalize RT-qPCR Cq values, this would not take into account group differences and might conceal slight differences between groups when normalizing results. While NormFinder does take into account a sample group such as placental sex, results using either GeNorm’s or NormFinder’s suggested genes did not provide the best reference gene to assess small mRNA level differences between samples. Here we have introduced a novel method of calculation for finding the best reference genes for normalization between groups. While this method is not perfect by any means, it is straightforward to use and can help pinpoint genes that have lower mRNA average variability between groups as well as the most stable in the pairwise variation method. The results presented in this study also highlights the importance of proper reference gene selection as well as the importance of not only relying blindly on software calculation results for such a critical part of the RT-qPCR technique. Thus, it is up to the researcher to verify and test differences between the mRNA levels of groups tested with raw RT-qPCR data.

## Materials and Methods

### Placental samples

Human term placentas (10 males/10 females; 38–41 gestation weeks) were obtained immediately after spontaneous vaginal deliveries from uncomplicated singleton pregnancies. Placentas were obtained with informed patient consent and approval of ethical committees at the CHUM-St-Luc Hospital (Montreal, QC) and INRS (Quebec, QC). Women having pathologies, smokers or under medication were excluded. All the experiments were performed according to ethical guidelines.

All samples were processes within 30 min of placental delivery. Placental tissue collection was realized using methods consistent with the recommendations^22^. Biopsies were taken, guided by a stereological grid, as described previously^23^. As the placenta is a heterogeneous tissue, it is necessary to reduce the sampling site-driven gene expression biases, which is achieved by multistage unbiased random sampling, thereby giving all sites equal possibility of selection. To maximize the representativeness of the extracted RNA, 5 tissue samples were collected from each placenta, using a stratified random sampling method, and then pooled for RNA extraction. The trophoblastic samples were then flash-frozen and kept at −80 °C until analysis. All tissue samples were then pooled and ground into powder using a mortar and pestle, kept at very low temperature by dry ice and liquid nitrogen. Placental tissue powders, 15 to 20 mg, were weighed using a 1.5 ml tube cooled in liquid nitrogen for RNA isolation. Such steps ensured that the placental samples were not thawed prior to RNA extraction.

### RNA isolation and cDNA synthesis

The RNA was isolated using the AllPrep DNA/RNA/Protein mini kit (Qiagen, Toronto, ON), after using Qiashredder spin columns (Qiagen) to further disrupt placental tissue, according to the manufacturer’s instructions. RNA concentration and purity were assessed using the ND-1000 Nanodrop Spectrophotometer (Thermo Scientific, Waltham, MA). RNA integrity was analyzed using the Experion automated electrophoresis system (Bio-Rad, Hercules, CA). Samples utilized had an RNA quality index (RQI) above 7.5. The cDNA was obtained using the iScript reverse transcription supermix for the RT-qPCR kit (Bio-Rad), following the manufacturer’s instructions, for a total of 500 ng of RNA. The samples were then diluted 1/20 in RNase Free Water and stored at −20 °C until further analysis.

### qPCR

The reference genes analyzed originated from PrimePCR Reference genes H96 and Reference Genes Plus H96 (Bio-Rad). These plates come with 14 different reference gene primers, lyophilized in wells (28 genes; Table 1), as well as controls for positive and negative PCR reaction, reverse transcription control and two RNA integrity controls. The primer sequence is not available for the genes in PrimePCR assays, but the validation data is available online for each gene. The reagent used for qPCR reaction was SsoAdvanced SYBR Green Supermix (Bio-Rad). The reaction was performed according to the manufacturer’s instructions, on a CFX96 real-time PCR detection system (Bio-Rad). Assays were performed in triplicate to control for technical errors.

**Table 1:** List of candidate reference genes included in the reference gene H96 and H96 plus prime PCR plates with RefSeq number.

Gene symbol	Name	RefSeq
ACTB	Beta Actin	NM_001101.3
ALAS1	Aminolevulinate, delta-, synthase 1	NM_000688.5
B2M	Beta-2-microglobulin	NM_004048.2
CDKN1A	Cyclin-dependent kinase inhibitor 1A (p21, Cip1)	NM_000389.4
G6PD	Glucose-6-phosphate dehydrogenase	NM_000402.4
GAPDH	Glyceraldehyde-3-phosphate dehydrogenase	NM_001256799.2
GUSB	Glucuronidase, beta	NM_000181.3
HBB	Hemoglobin, beta	NM_000518.4
HMBS	Hydroxymethylbilane synthase	NM_001024382.1
HPRT1	Hypoxanthine phosphoribosyltransferase 1	NM_000194.2
HSP90AB1	Heat shock protein 90 kDa alpha (cytosolic), class B member 1	NM_001271969.1
IPO8	Importin 8	NM_001190995.1
LDHA	Lactate dehydrogenase A	NM_001135239.1
NONO	Non-POU domain containing, octamer-binding	NM_001145408.1
PGK1	Phosphoglycerate kinase 1	NM_000291.3
PPIA	Peptidylprolyl isomerase A (cyclophilin A)	NM_001300981.1
PPIH	Peptidylprolyl isomerase H (cyclophilin H)	NM_006347.3
PSMC4	Proteasome (prosome, macropain) 26S subunit, ATPase, 4	NM_006503.3
PUM1	Pumilio RNA-binding family member 1	NM_001020658.1
RPL13A	Ribosomal protein L13a	NM_001270491.1
RPL30	Ribosomal protein L30	NM_000989.3
RPLP0	Ribosomal protein, large, P0	NM_001002.3
RPS18	Ribosomal protein S18	NM_022551.2
SDHA	Succinate dehydrogenase complex, subunit A, flavoprotein (Fp)	NM_001294332.1
TBP	TATA box binding protein	NM_001172085.1
TFRC	Transferrin receptor (p90, CD71)	NM_001128148.1
UBC	Ubiquitin C	NM_021009.6
YWHAZ	Tyrosine 3-monooxygenase/tryptophan 5-monooxygenase activation protein, zeta	NM_001135699.1

### Data analysis

To analyze the reference genes’ mRNA expression stability, the qBase + software package (BioGazelle, Zwijnaarde, Belgium) was used to obtain GeNorm results. The qBase + software package calculates gene expression stability (GeNorm M), as well as the variation from using n reference gene or n + 1 reference genes to evaluate the lowest number of genes required for accurate normalization (GeNorm V). Statistical analyses were carried out using PRISM 5.0 (Graphpad software, La Jolla, CA). Variance was tested between sexes and normal sample distribution was also assessed, prior to using Student’s t-test to evaluate the difference in mRNA level between male and female placentas.

## Electronic supplementary material

Supplementary Information
