# Supplementary figures and images for: A simple method to assess group difference in RT-qPCR reference gene selection using GeNorm: The case of the placental sex
Source: Sci Rep. 2017 Dec 5;7:16923. doi: 10.1038/s41598-017-16916-y (PMC5717228; doi:10.1038/s41598-017-16916-y)

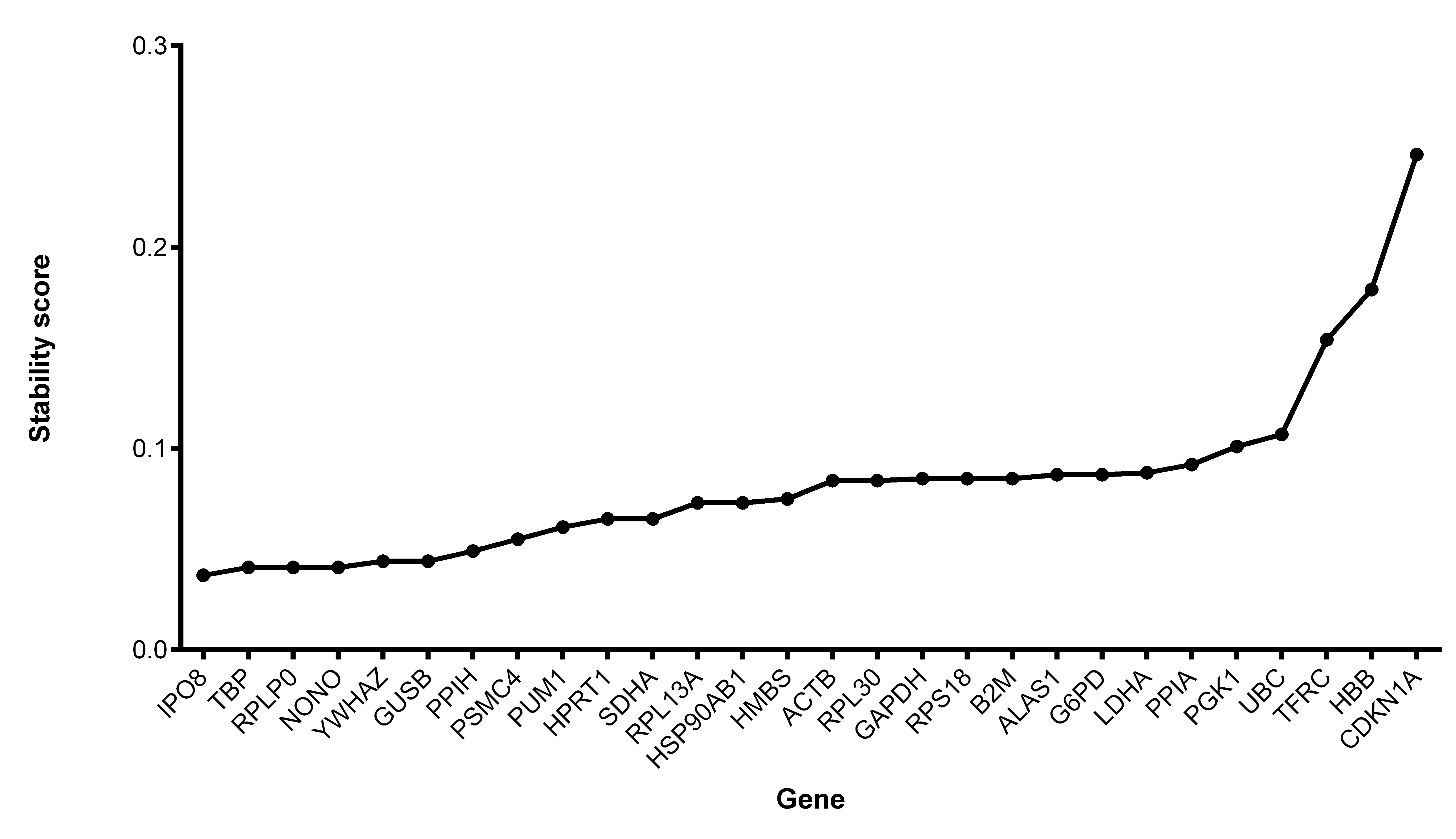

Supplement: Supplementary file 1 — Supplementary Information [file 41598_2017_16916_MOESM1_ESM.jpg]
